# Advanced platelet-rich fibrin promotes healing of induced corneal ulcer in donkeys (*Equus asinus*)

**DOI:** 10.1038/s41598-023-48933-5

**Published:** 2023-12-09

**Authors:** Omar H. Hosny, Mahmoud Abd-Elkareem, Magda M. Ali, Ahmed F. Ahmed

**Affiliations:** 1 Department of Surgery, Anesthesiology and Radiology, Faculty of Veterinary Medicine, Assiut University, Assiut, 71526 Egypt; 2 Department of Cell and Tissues, Faculty of Veterinary Medicine, Assiut University, Assiut, 71526 Egypt

**Keywords:** Cell growth, Cell proliferation, Ciliogenesis, Differentiation, Regeneration

## Abstract

Ulcerative keratitis is a common disease in horses which may cause blindness. To prevent secondary bacterial and fungal infections and promote quick re-growth of the epithelial layer, different treatment approaches have been employed. This study aimed to examine the effects of advanced platelet-rich fibrin (A-PRF) gel on the healing process of experimentally induced corneal ulcers in donkeys. Nine healthy adult donkeys were used for the study. The donkeys were divided into two groups: the control group, where no medication was applied to the corneal ulcer, and the A-PRF gel group, where A-PRF gel was applied once a day on specific days after ulcer induction. The healing process was evaluated through various examinations and analyses. The results demonstrated that the A-PRF gel group showed significant improvement in the corneal ulcer area, with epithelial and stromal regeneration. At day 35, about 60% of the A-PRF group showed negative fluorescein uptake. Additionally, fewer complications were observed during the healing process compared to the control group. In conclusion, A-PRF gel is an important and safe therapeutic option for controlling ocular surface infection and promoting corneal healing. We recommend using A-PRF gel as an alternative approach, avoiding eyelid suturing, and minimizing corneal irritation.

## Introduction

Corneal ulcers are a significant concern in the equine industry and for veterinarians due to the unique anatomy of horse's eyes^1–5^. Ulcerative keratitis, characterized by inflammation and damage to the cornea, can occur as a result of infections caused by microorganisms from the environment or the ocular surface. These infections can lead to the infiltration of immune cells and the production of enzymes that can cause the cornea to melt^1,6,7^. Complex corneal ulcers, which involve deep layers of the cornea and are often associated with infection, are particularly challenging^8,9^.

Various treatment approaches have been used to prevent secondary infections and promote rapid healing of corneal ulcers, but the optimal drug therapy is still uncertain1. Medical therapy is often the first choice, as differentiation between simple superficial corneal erosions and superficial non-healing corneal ulcers may be difficult at the onset of ocular problems. The medical treatments generally give disappointing results with superficial non-healing corneal ulcers while the absence of response after a while may be a sign of the presence of such ulcers^10^. The goals of medical therapy for superficial corneal ulcers are to reduce contamination and prevent bacterial colonization of the exposed corneal stroma until re-epithelialization has occurred^11^. A soft contact lens can be used to protect the cornea. In an animal that was medically treated for an indolent ulcer, the affected eye became significantly less painful as soon as the soft contact lens was applied. The contact lens allowed the animal to undergo training during the time of treatment. When loosened epithelial margins are present, debridement is advised using a cotton tip or small curette^12^. Superficial debridement of the cornea (Keratectomy) in conjunction with temporary tarsorrhaphy, third eyelid flap, or conjunctival flap, can be used with unmedical treatable cases^13^. Recent advancements in regenerative therapies aim to restore damaged tissues and address ophthalmological conditions^14^. One of the recent interesting regenerative therapies is amniotic membrane transplantation (AMT) and the use of recombinant growth factors. However, the results obtained after the application of AMT, but the costly manufacturing, and the seldom clinical results, make it obligatory to find other therapeutic ways for corneal regeneration^15–18^. Matrix regenerating agents (RGTA) are recent topical agents that show encouraging results in promoting tissue healing and regeneration, including cases of neurotrophic ulcers, corneal dystrophies, or postsurgical conditions^19,20^. Recently, drops coming from blood derivatives have also been described as a treatment for ophthalmology disorders, including persistent corneal epithelial defects^18^. Platelet concentrates, derived from a patient's blood, contain growth factors, fibrin, and sometimes leukocytes, which are collected and processed into a clinically functional form^21,22^. These concentrates can be in liquid form for distillation or injection, or gel form for application to wounds or injured areas to promote tissue regeneration^22–25^. Recently, a complete classification of platelet concentrate technologies depending on their leukocyte content and fibrin architecture has been proposed^26^. One specific type of platelet concentrate is platelet-rich fibrin (PRF), which is an autologous fibrin-based membrane, matrix, or scaffold ^21,25,27–30^.

## Aim of work

The impact of using autologous advanced platelet-rich fibrin gel on the recovery of artificially-induced corneal ulcers in donkeys (*Equus asinus*).

## Material and methods

### Ethical approval

This study was conducted as a brief prospective investigation at the Veterinary Teaching Hospital, Faculty of Veterinary Medicine, Assiut University, Assiut, Egypt, specifically in the Department of Surgery, Anesthesiology, and Radiology. The procedures of this study were approved by the National Ethics Committee of the Faculty of Veterinary Medicine, University of Assiut, Assiut, Egypt, in accordance with both Egyptian law and the Animal Welfare Standards for the Care and Use of Animals in Research and Education set by the OIE. The research was carried out in adherence to the guidelines and regulations outlined in the ARRIVE guidelines (https://arriveguidelines.org).

### Animals

The study involved a total of nine donkeys (Equus asinus), consisting of four males and five non-pregnant, non-lactating females. The donkeys were selected based on their age, ranging from 7 to 8 years with an average of 7.4 years, and their body weights, ranging from 100 to 130 kg with an average of 115 kg. Prior to the study, the donkeys underwent an eye examination to ensure they had no pre-existing eye deformities. Throughout the study, the donkeys were housed in well-equipped stalls and provided with a balanced ration twice a day, along with access to green forage. They were also given unrestricted access to water.

### Ophthalmologic examination

The eyes were assessed for their normal shape, presence of any discharge, position and movement of the eyeballs, reflexes of the eyelids and cornea, as well as the threat response. In a dimly lit room, the pupil's response to light, condition of the eyelids, nictitans, conjunctiva (both bulbar and palpebral), cornea, iris, and lens were thoroughly examined^31^.

### Anesthesia

To sedate the animals, they were given an intravenous (IV) injection of 1.1 mg/kg xylazine hydrochloride 2% (Xyla-Ject, ADWIA Co., SAE, Egypt) and 2.2 mg/kg ketamine hydrochloride 5% (Ketamine, Sigma- tec Pharmaceutical Industries, SAE, Egypt). Additionally, three drops of benoxinate hydrochloride 0.4% (Benox, Sterile Ophthalmic Solution, Egyptian Int. Pharmaceutical Industries Co., Egypt) were applied to the corneal surface as a surface analgesia.

### Induction of a centric corneal ulcer (Fig. 1)

**Figure 1:**
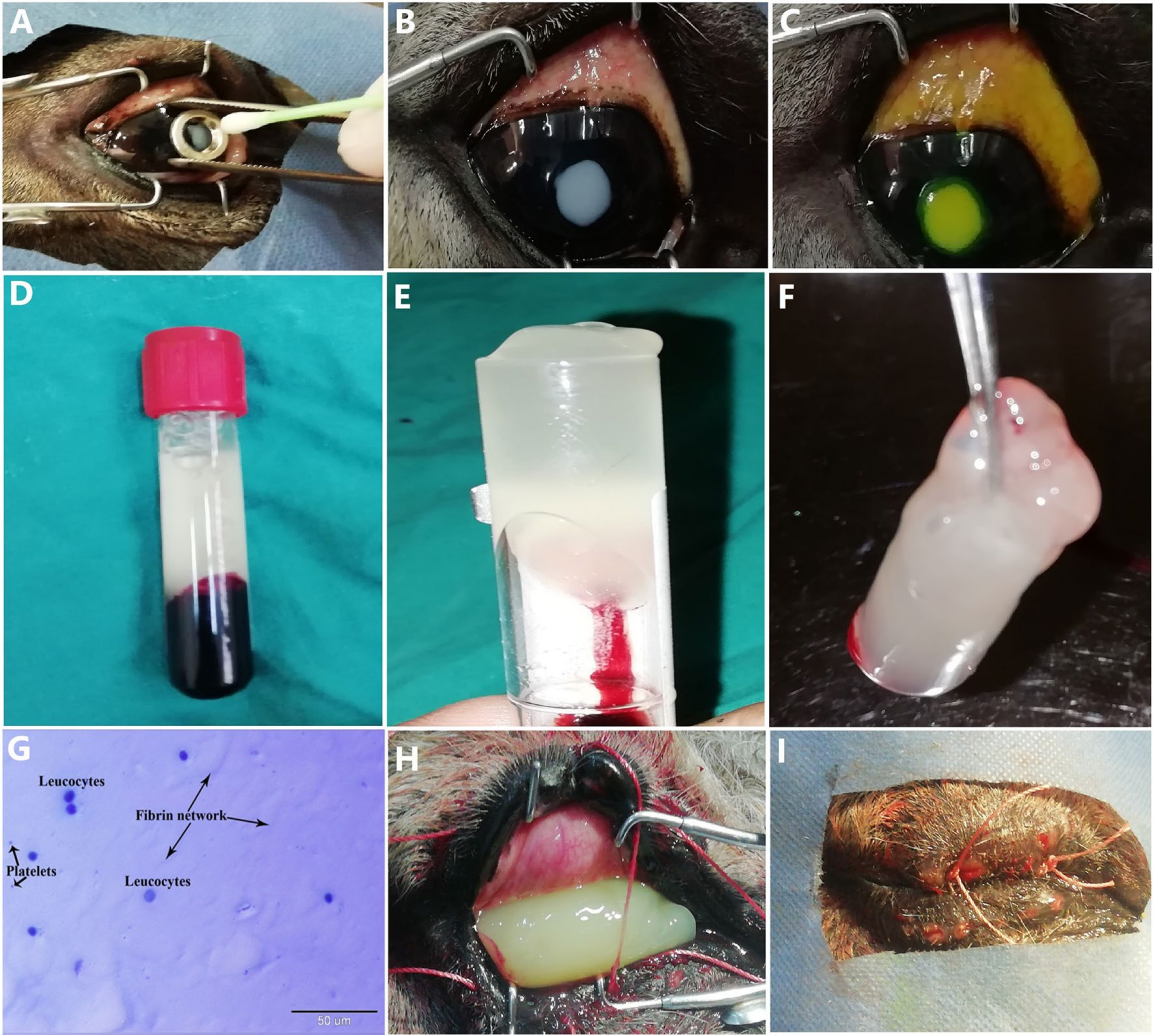
Method of inducing corneal ulcers; a cotton swab dipped in 1N NaOH was applied to the corneal surface, after using a sterile stainless steel washer as a template (**A**), a central corneal ulcer was now induced and X-rayed with a ruler as a yardstick (**B**) and fluorescein staining of the ulcer (**C**). Preparation and application of the A-PRF gel; the A-PRF gel was formed in the Vacutainers after centrifugation (**D**), the gel was then brought out with sterile forceps (**E**), the finished gel after cutting with scissors (**F**), stained smear of the A-PRF gel examined by a light microscope (**G**), the A-PRF gel was applied to the corneal surface after ulcer induction (**H**), and two interrupted skin sutures were placed to hold the gel in place (**I**).

The donkey was positioned on its side after anesthesia, with the operated eye placed on top. To induce a centric corneal ulcer with a diameter of 6 mm, a solution of 1N sodium hydroxide (NaOH) was used^32,33^. A sterile cotton swab with a diameter of 4 mm was soaked in the NaOH solution for 5 s. The eye was opened using an eyelid dilator, and the swab was placed on the corneal surface for 60 s^34^. To ensure a uniform circular ulcer, a sterile stainless steel washer with a 6 mm inner diameter was used as a template. The excess NaOH was then rinsed off by irritating the eye with 20 mL of physiological saline^35^. The donkeys were randomly divided into two main groups: the control group (A) consisting of three donkeys, where no drugs were applied to the corneal ulcer throughout the study, and the advanced platelet-rich fibrin gel (A-PRF gel) group (B) consisting of six donkeys, where A-PRF gel volume which obtained from centrifugation of 10 ml blood of own animal without any anticoagulants was applied topically over the cornea (in the cal de sac) once daily on days 0, 2, 4, 6, 9, 13, 20 , and 27 after corneal ulcer induction. To ensure the gel remained in contact with the corneal surface, the lid fissure was closed using two interrupted horizontal silk sutures (#0) that went through the skin of the upper and lower eyelids (skin to skin). This was done to keep the A-PRF gel in place (see Fig. 1).

### Preparation of the A-PRF gel (Fig. 1)

A 10 ml sample of the donkey's own venous blood was collected from the jugular vein using a sterile 10 ml syringe and an 18G1.1/2 hypodermic needle. The blood was immediately placed in a sterile glass-based vacuum tube. The tube was then centrifuged at 1500 rpm for 14 min using a CENTRIFUGE PLC SERIES, Model: PLC-05, SN.818555, Gemmy Industrial Corp., Taiwan. This process resulted in the formation of three layers: platelet-poor plasma (PPP) on the top, red blood cells at the bottom, and the A-PRF gel or clot in the middle^32,36^**.** To use the A-PRF gel, the clot was carefully removed from the tube using sterile forceps, and the layer of red blood cells was trimmed away with sterile scissors to obtain a clean A-PRF gel.

### Evaluation criteria

All animals were closely monitored and assessed on specific days (2, 4, 6, 9, 13, 20, 27, and 35) after the corneal ulcers were induced. To conduct eye examinations and capture photographs, the animals were sedated using an intravenous injection of 1.1 mg/kg xylazine hydrochloride 2%. Additionally, retrobulbar and auriculo-papebral nerve blocks were performed using 20 ml and 5 ml of 2% lidocaine hydrochloride, respectively. The evaluation process included clinical and external ophthalmologic examinations, fluorescein staining, assessment of ulcer healing, and histopathology.

#### Clinical examination

The donkeys were observed and evaluated for their activity levels, resumption of eating and drinking, and signs of pain for a period of 3–5 days after waking up from anesthesia. Physiological measurements such as body temperature (BT), heart rate (HR), and respiratory rate (RR) were recorded both before the surgery and after the donkeys had fully recovered from anesthesia.

#### External ophthalmologic examination

The cornea and conjunctiva were visually inspected for signs of swelling, excessive tearing, discharge, and eyelid spasms. Using a binocular loupe and a focused light source, the presence of corneal opacity, neovascularization, pigmentation, and conjunctivitis were also examined. These assessments were conducted on days 2, 4, 6, 9, 13, 20, 27, and 35 following the induction of corneal ulcers. Additionally, the healing progress of the ulcers and the formation of scar tissue were evaluated.

#### Fluorescein staining test

To assess the presence of corneal ulceration, a 2% fluorescein solution was applied to the eye on days 2, 4, 6, 9, 13, 20, 27, and 35. The solution was left in the eye for one minute before being flushed out with sterile normal saline. The eye was then examined for any remaining stain using a pen light and binocular loupe^37^.

#### Ulcer healing monitoring

Photographs of the eye were taken twice during the study: once after conducting an external eye examination and again after fluorescein staining. These photographs were captured on days 2, 4, 6, 9, 13, 20, 27, and 35 using a digital camera. Prior to capturing the images, the eye was flushed with normal saline, and a standard ruler was placed as close as possible to the ulcer. To assess the rate of ulcer healing, the surface areas of the corneal ulcer were measured in square centimeters using Image J software (version 4.48v, National Institutes of Health, USA) and analyzed^32^.

#### Histopathological examination

Histopathological examination was performed after donkeys were treated according to the protocol^38^ by intravenous injection of xylazine hydrochloride (1.1 mg/kg) followed by rapid intravenous injection of thiopental sodium (thiopental sodium 1 g vial, EPICO, Egypt) at a dose of 35 mg/kg; the corneas of the donkeys were collected and fixed in 10% neutral buffered formalin. The fixed samples were then dehydrated, clarified, and embedded in paraffin wax. Thin sections (5 µm) were cut from the paraffin blocks and stained using various histological techniques. Hematoxylin and eosin (H&E) stain was used for general histological examination^39^, periodic acid-Schiff (PAS) technique was employed to detect neutral mucopolysaccharides^40^, Crossmons trichrome technique was used to stain corneal collagen fibers^41^, and Picro-Sirius red technique was utilized to differentiate between mature and immature corneal collagen^42^. The stained sections were observed under an Olympus BX51 microscope, and images were captured using an Olympus DP72 camera attached to the microscope.

### Statistical analysis

The data were analyzed using the GLM procedure of SAS (SAS Institute, 2009) and expressed as mean ± SE. Significant treatment effects were determined using ANOVA, and differences between the least squares means were assessed using Duncan's multiple range test. Statistical significance was considered at a level of P < 0.05.

## Results

### Clinical examination

On the day of ulcer induction, the donkeys exhibited signs of pain such as restlessness, head droop, isolation, and decreased food intake. However, by the fourth day after ulcer induction, most of the donkeys had returned to normal food consumption and the head droop had resolved in all but two of them. These two donkeys continued to show head droop and decreased activity until the fourth postoperative day. However, by the fifth day of the study, all donkeys had returned to normal activity and performance. The physiological parameters, including heart rate (HR), respiratory rate (RR), and body temperature (BT), remained within the normal range throughout the study, and there was no significant difference in these parameters before and after ulcer induction (Table 1).

**Table 1:** Values (mean ± SE) of physiological parameters of donkeys before induction of corneal ulceration and after recovery from anesthesia.

Group	Parameter	Preoperative	After recovery
Control (A)	BT (°C)	38.93 (0.3)	38.87 (0.3)
	HR (beat/min)	102.3 (6.1)	96.1 (3.6)
	RR (breath/min)	74.3 (4.2)	69.8 (3.6)
A-PRF-gel (B)	BT (°C)	38.8 (0)	38.8 (0.3)
	HR (beat/min)	110 (3.6)	101.3 (2.9)
	RR (breath/min)	81.7 (7.4)	77.6 (6.2)

*BT* body temperature, *HR* heart rate, *RR* respiratory rate.

### External eye examination

#### Control group (A): (Fig. 2)

**Figure 2:**
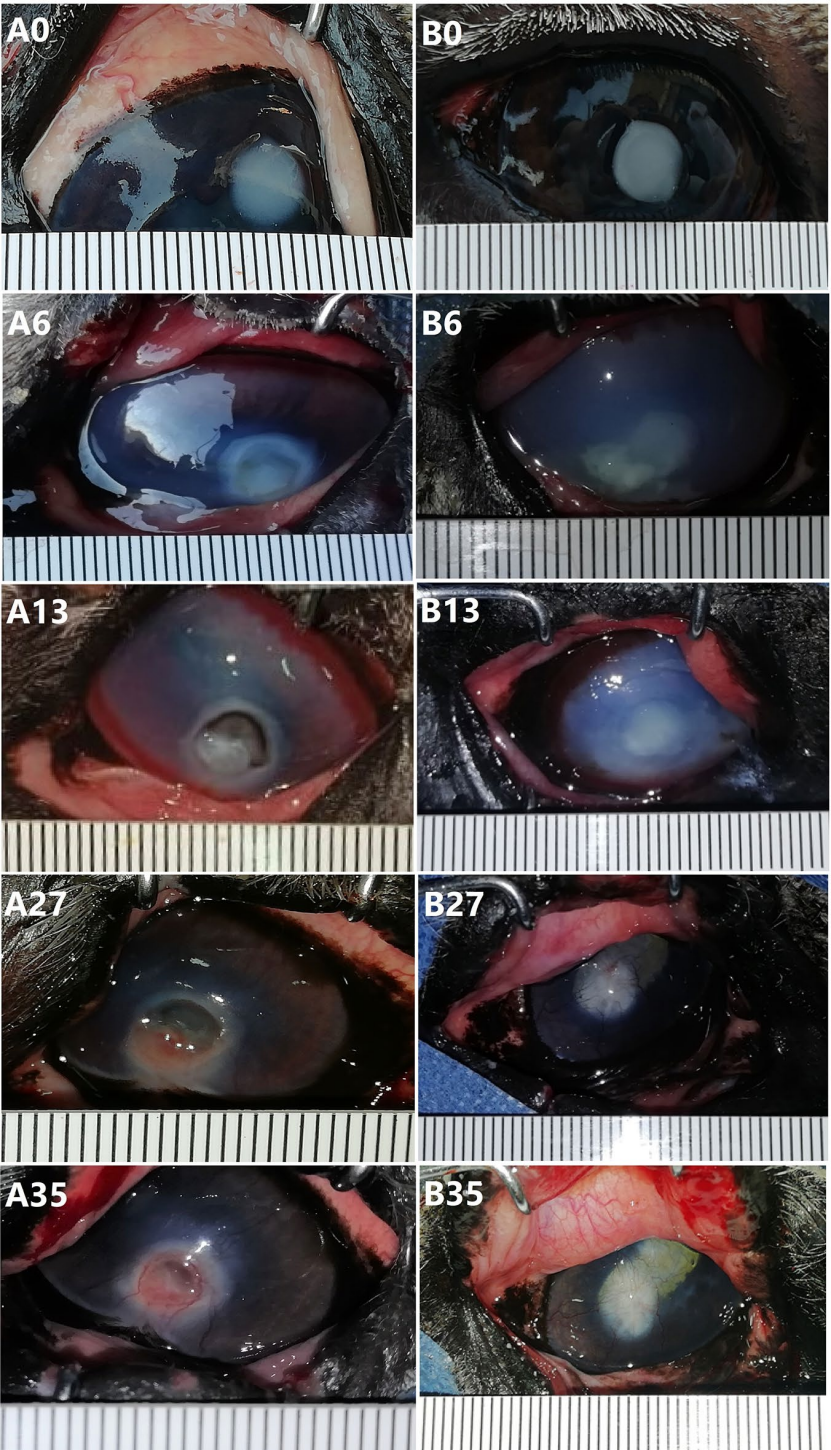
Serial photos of the follow-up of the induced corneal ulceration in the control and A-PRF gel group at days 0, 6, 13, 27 and 35 in donkeys.

From the second day after ulcer induction until day 35, blepharospasm (eye twitching) was observed in all donkeys except one, which resolved by day 20. Epiphora (excessive tearing) and corneal opacity were present on the second day after ulcer induction and gradually decreased from day 20 to day 35. Corneal vascularization (formation of new blood vessels) varied in severity, with some donkeys showing superficial neovascularization and others showing deep neovascularization. Overall, corneal vascularization decreased gradually from day 20 to day 35. Two donkeys in the control group had associated conjunctivitis, which resolved in one donkey by day 13.

#### Gel-treated group (B): (Fig. 2)

Blepharospasm and epiphora were observed in all donkeys in this group, except for two donkeys, from day 2 until the end of the study (day 35). Eyelid swelling started on the second day of ulceration and resolved on the ninth day in three donkeys, and on the 27th day in the other three donkeys. Corneal vascularization varied in severity, with some donkeys showing superficial neovascularization and others showing deep neovascularization. Neovascularization peaked on day 13 and started on day 2 in four animals and on day 4 in two animals. Conjunctival congestion was present in all animals from the second day of the study and began to resolve on the ninth day in two animals and the 20th day in four animals.

### Results of fluorescein staining (Fig. 3)

**Figure 3:**
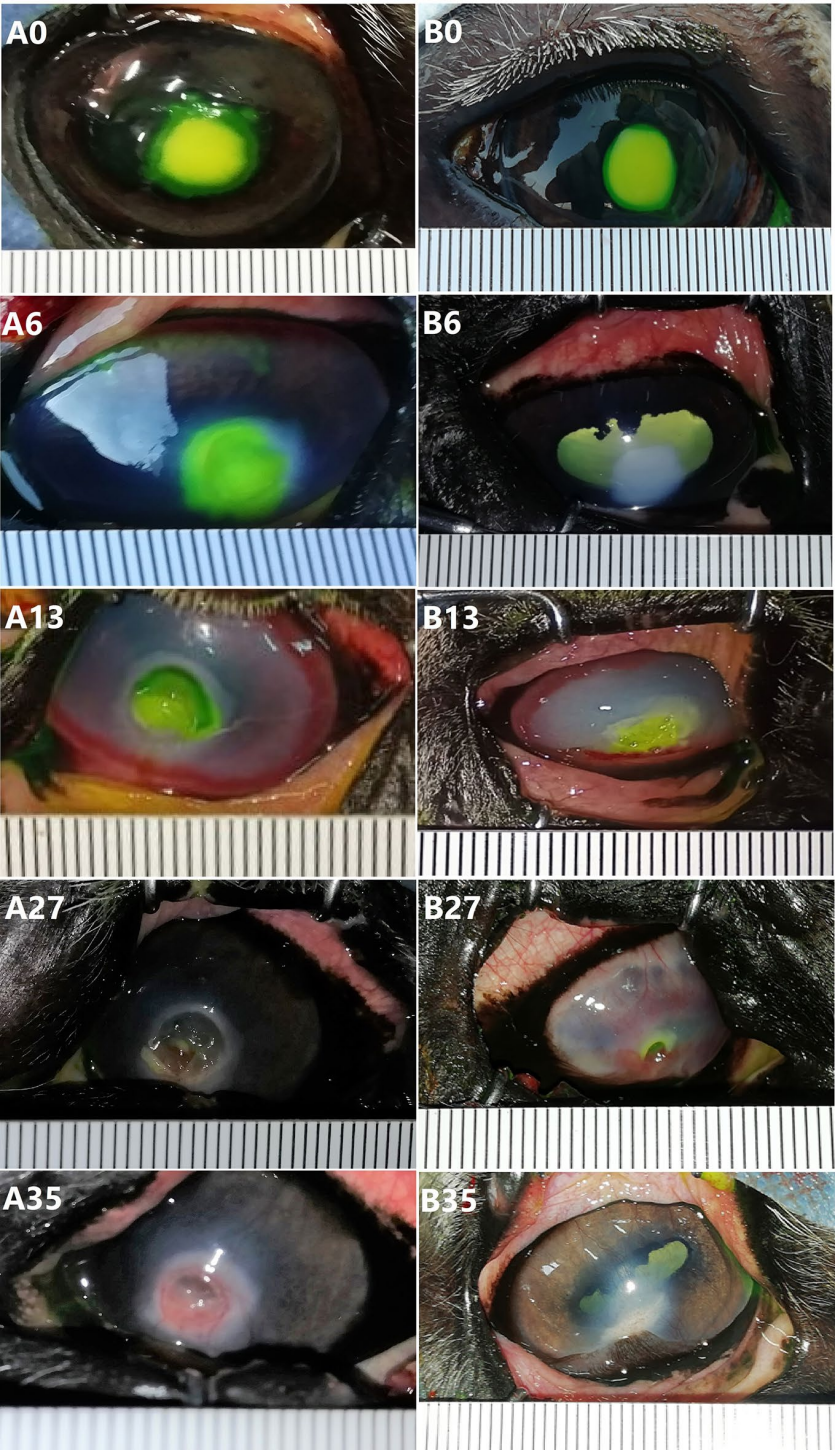
Serial photos of follow-up of induced corneal ulceration after fluorescein staining in control and A-PRF gel group at days 0, 6, 13, 27 and 35 in donkeys.

In the control group (A), two donkeys showed a disappearance of fluorescein uptake on day 20, while one donkey showed a disappearance on day 35. In the A-PRF gel group (B), four donkeys had a disappearance of fluorescein uptake on day 35, one donkey on day 9, and one donkey had a reuptake on day 6 followed by a disappearance on day 9 and again on day 13.

### Ulcer healing results

**Figure 4:**
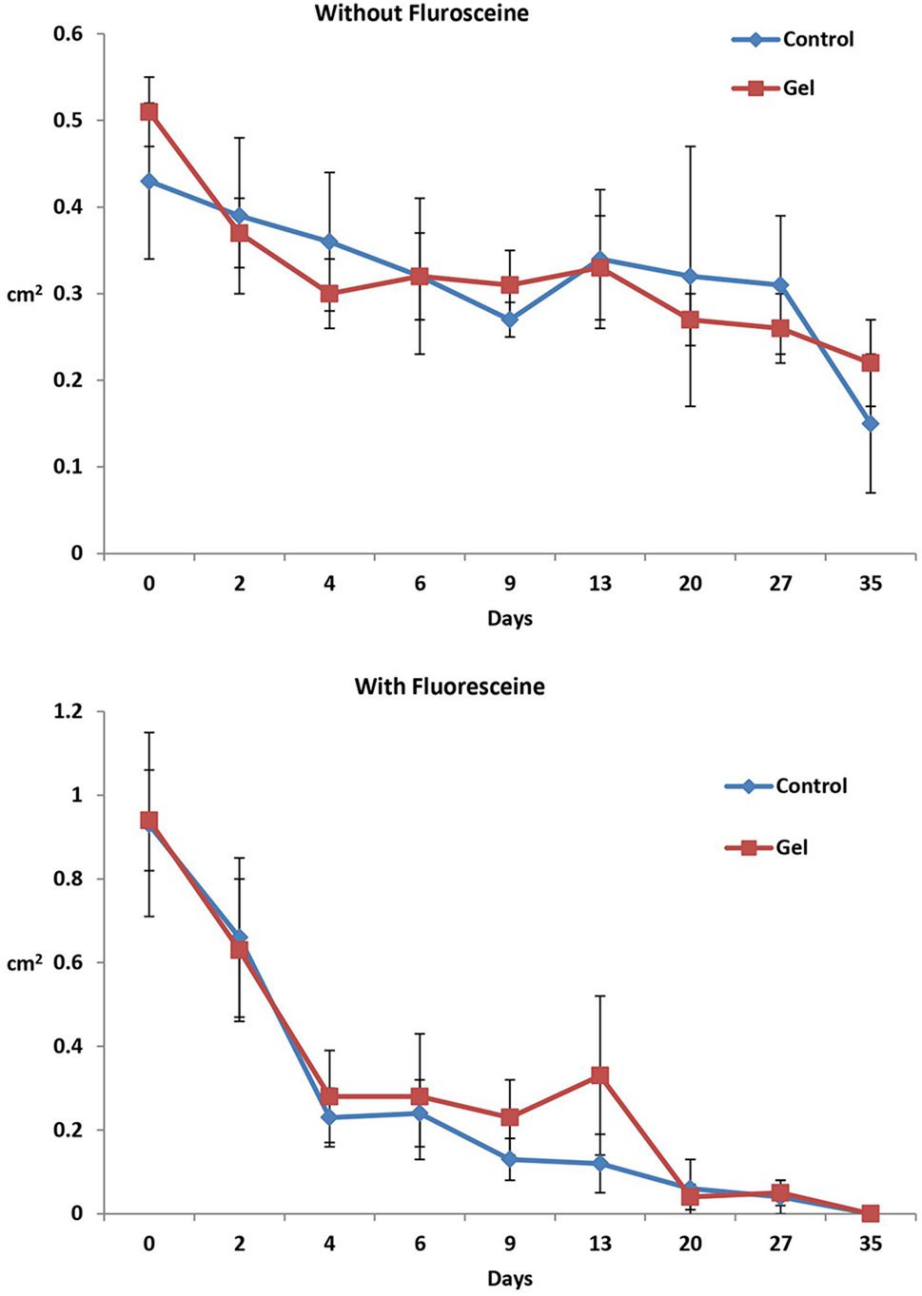
Mean (SE) surface area (in cm^2^) of corneal ulceration without fluorescein (top) and with fluorescein (bottom) in the control group (n = 3) and the A-PRF gel group (n = 6).

In group (A), the average size of the ulcer (measured in cm2) at day 35 was significantly reduced (P < 0.041) compared to the initial measurement at day zero (Table 2, Fig. 4). Similarly, in group (B), the average ulcer size at days 2, 4, 6, 9, 13, 20, 27, and 35 showed a significant decrease (P < 0.0001) compared to the initial measurement at day zero (Table 2, Fig. 4). The results of image analysis after fluorescein staining also indicated a significant decrease in the average ulcer size at days 4, 6, 9, 13, 20, 27, and 35 compared to day zero in both group (A) and group (B) (P < 0.0001) (Table 2, Fig. 4).

**Table 2:** Values (mean ± SE) of the surface area (cm^2^) of the corneal ulceration and fluorescein-stained corneal ulceration of the donkeys as measured by the software (Image J).

Day	Non-stained corneal ulcers		Fluorescein-stained corneal ulcers	
	Group (A) (*n* = 3)	Group (B) (*n* = 6)	Group (A) (*n* = 3)	Group (B) (*n* = 6)
0	0.43 (0.09)^a^	0.51 (0.04)^a^	0.93 (0.22)^a^	0.94 (0.12)^a^
2	0.39 (0.09)^a^	0.37 (0.04)^b^	0.66 (0.19)^aA^	0.63 (0.17)^aA^
4	0.36 (0.08)^a^	0.30 (0.04)^b^	0.23 (0.07)^b^	0.28 (0.11)^b^
6	0.32 (0.09)^a^	0.32 (0.05)^b^	0.24 (0.08)^b^	0.28 (0.15)^b^
9	0.27 (0.02)^a^	0.31 (0.04)^b^	0.13 (0.05)^b^	0.23 (0.09)^b^
13	0.34 (0.08)^a^	0.33 (0.06)^b^	0.12 (0.07)^b^	0.33 (0.19)^b^
20	0.49 (0.15)^aA^	0.27 (0.03)^bB^	0.06 (0.07)^b^	0.04 (0.03)^b^
**27**	0.31 (0.08)^a^	0.26 (0.04)^b^	0.04 (0.04)^b^	0.05 (0.03)^b^
35	0.15 (0.08)^b^	0.22 (0.05)^b^	0 (0)^bA^	0 (0)^bA^

Values of different superscript small letters over time (within column) and capital letters between groups (within row) are significantly different at p < 0.05.

**Figure 5:**
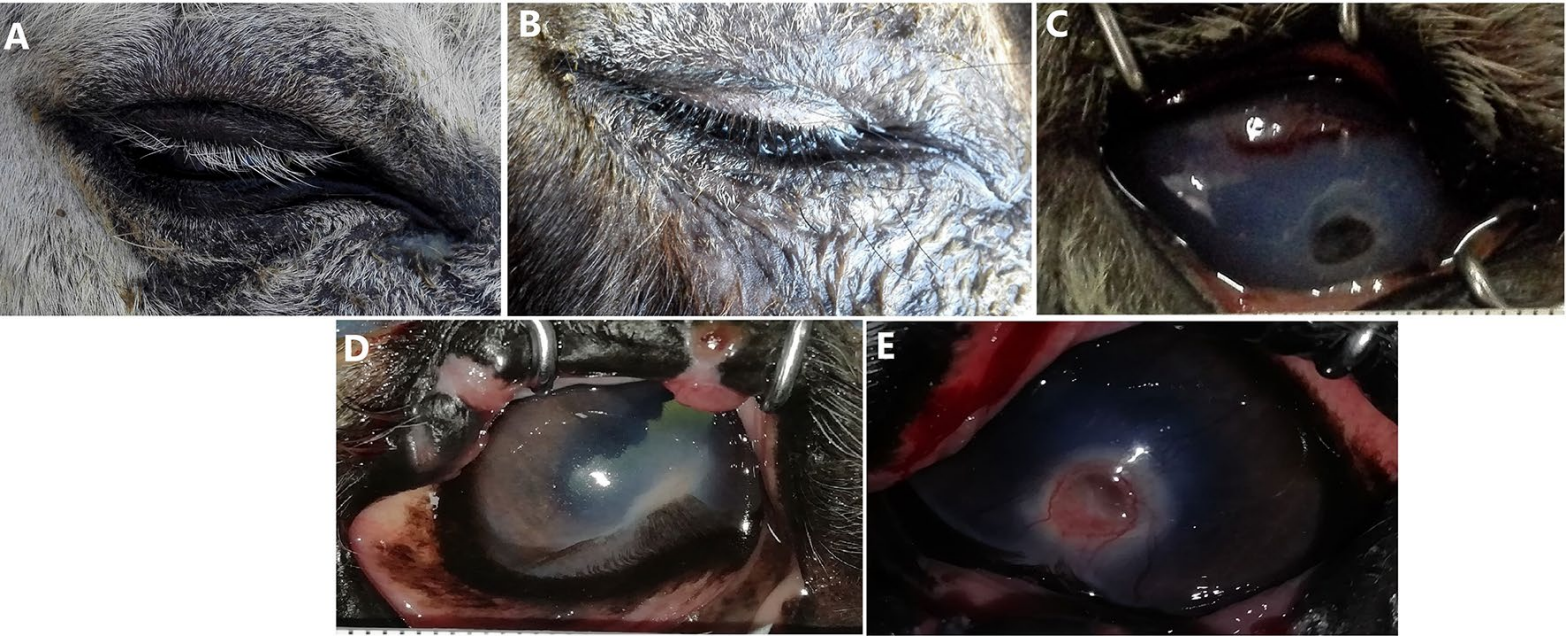
Complications recorded during the follow-up period of induced corneal ulceration in donkeys; eyelid swelling and blepharospasm (**A**), epiphora (**B**), anterior synechia (**C**), pigmented keratitis (**D**), pigmented keratitis with scarring (**E**).

During the study, several complications were observed (Fig. 5). These included pigment keratitis, which was seen in one donkey from group (A) and four donkeys from group (B) starting from day 20 until the end of the study. Additionally, one donkey from the group (B) developed anterior synechia on day 20.

### Results of the histopathological examination

**Figure 6:**
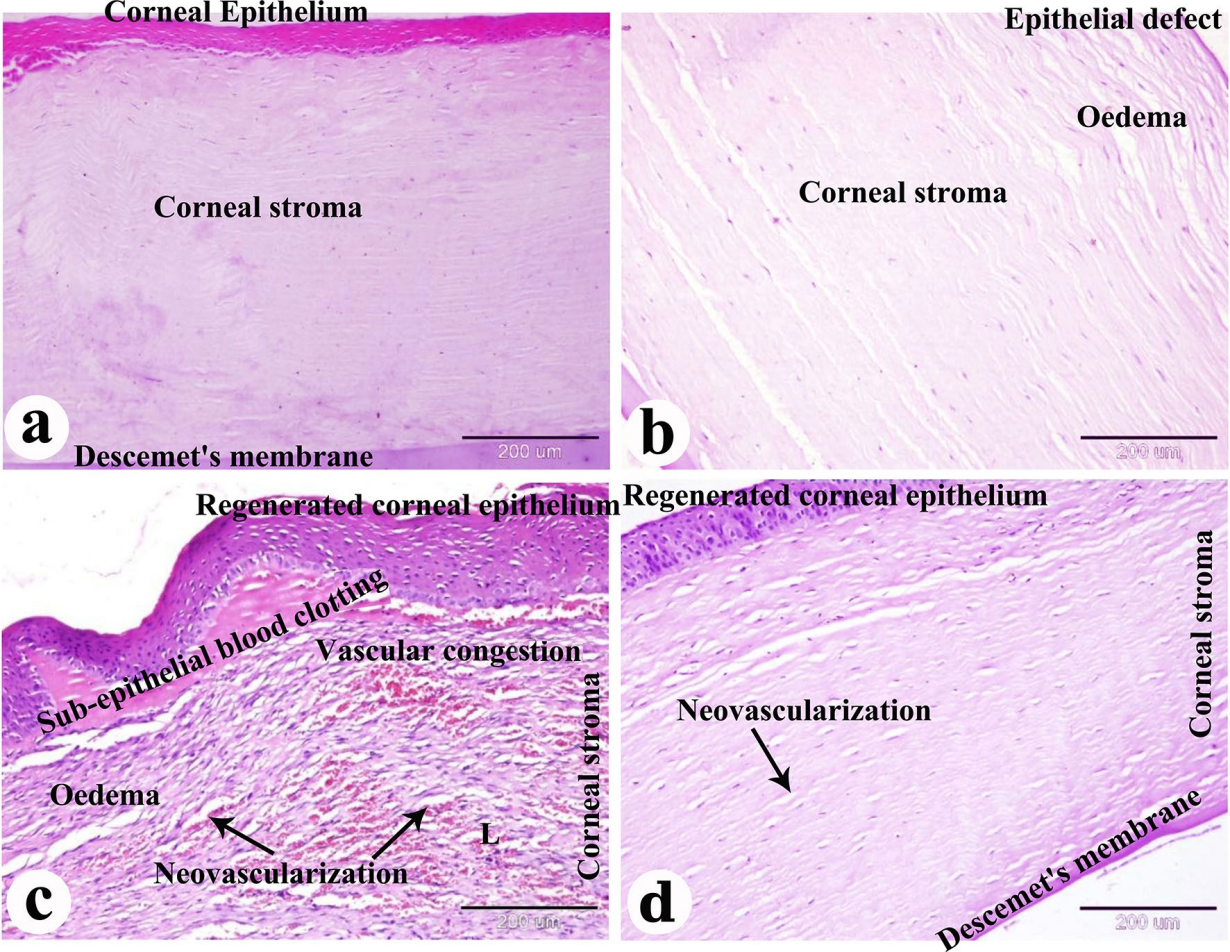
Photomicrograph of paraffin sections in donkey cornea. (**a**) The negative control group shows the normal histological structure of the cornea. (**b**) Day 0 group with induced corneal ulcer showing epithelial defect and edema in the corneal stroma. (**c**) The positive control group shows regenerated corneal epithelium, subepithelial blood coagulation, edema, leukocyte infiltration (L), vascular congestion and neovascularization in the corneal stroma. (**d**) The PRF gel panel shows regenerated corneal epithelium and neovascularization in the corneal stroma. Original enlargement; ×100, scale bar = 200 µm, hematoxylin and eosin stain.

**Figure 7:**
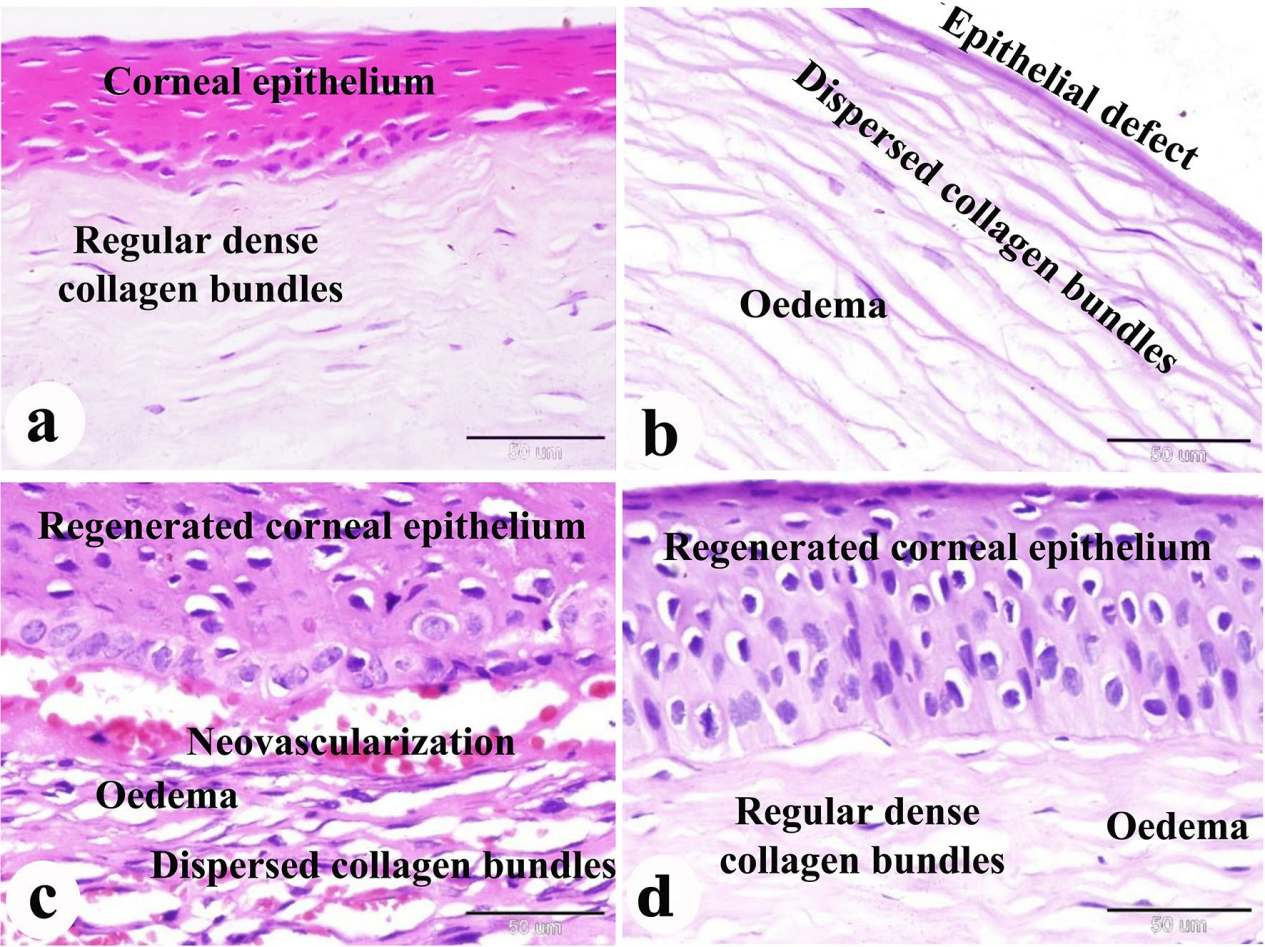
Photomicrograph of paraffin sections in donkey cornea. (**a**) The negative control group shows the normal histological structure of the cornea. (**b**) Day 0 group with induced corneal ulcer showing an epithelial defect, dispersed collagen bundles and edema in the corneal stroma. (**c**) The positive control group shows regenerated corneal epithelium and dispersed collagen bundles, neovascularization, edema in the corneal stroma. (**d**) The PRF gel group shows regenerated corneal epithelium and regularly dense collagen bundles, edema in the corneal stroma. Original enlargement; ×400, scale bar = 50 µm, hematoxylin and eosin stain.

**Figure 8:**
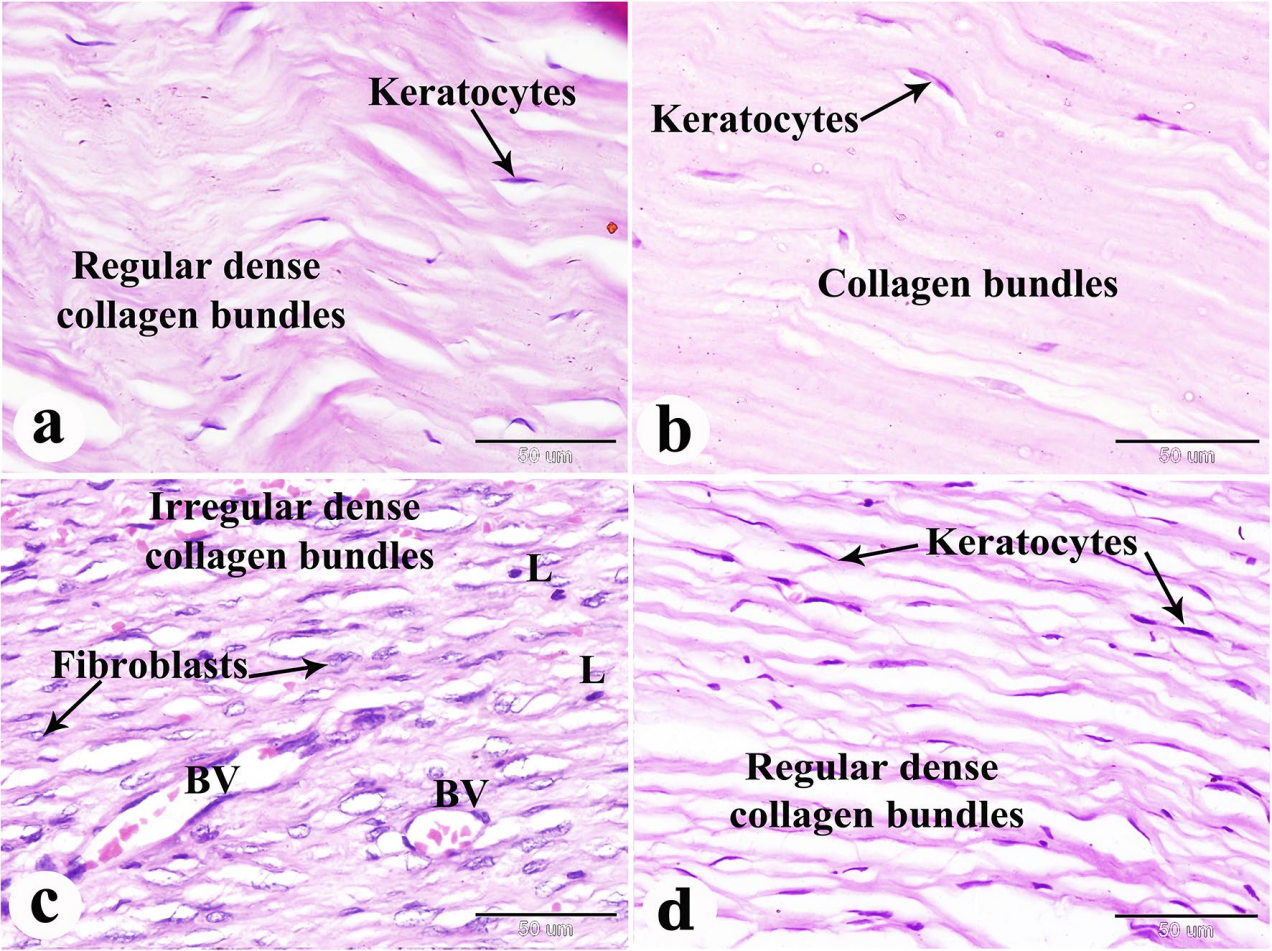
Photomicrograph of paraffin sections in donkey cornea. (**a**) The negative control group shows the normal histological structure of the cornea; numerous keratocytes between the dense regular collagen bundles. (**b**) Group with induced corneal ulcer on day 0 showing numerous keratocytes between the collagen bundles. (**c**) The positive control group showing numerous fibroblasts between the irregular dense collagen bundles, neovascularization (BV) and leukocyte infiltration (L). (**d**) The PRF gel panel with numerous keratocytes between the dense regular collagen bundles. Original enlargement; ×400, scale bar = 50 µm, hematoxylin and eosin stain.

**Figure 9:**
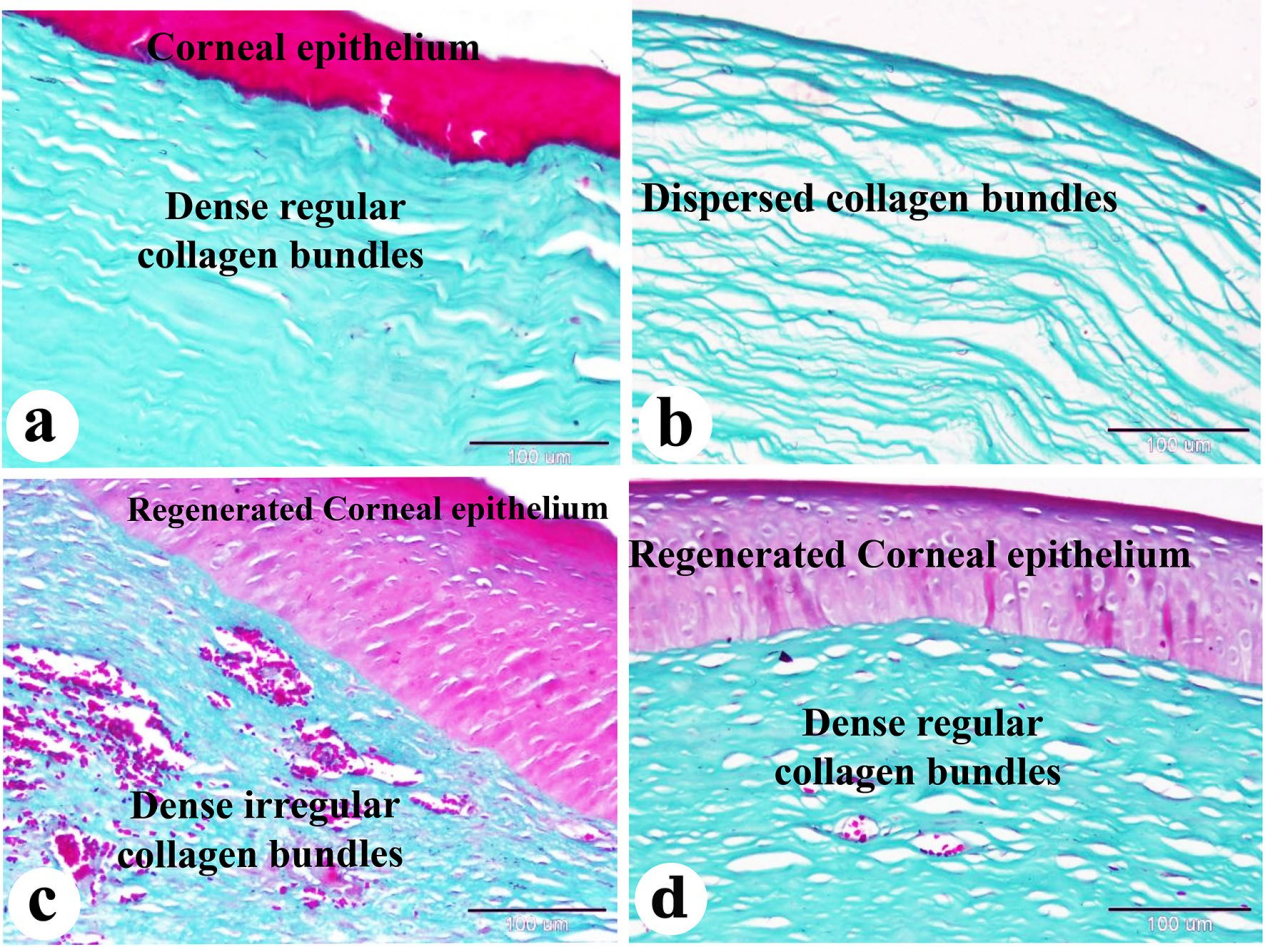
Photomicrograph of paraffin sections in donkey cornea. (**a**) The negative control group shows the normal histological structure of the cornea; Corneal epithelium and dense regular collagen bundles. (**b**) Day 0 group with induced corneal ulcer showing dispersed collagen bundles. (**c**) The positive control group showing regenerated corneal epithelium and dense irregular collagen bundles. (**d**) The PRF gel group shows regenerated corneal epithelium and dense regular collagen bundles. Original enlargement; ×200, scale bar = 100m, Crossmons trichrome staining.

**Figure 10:**
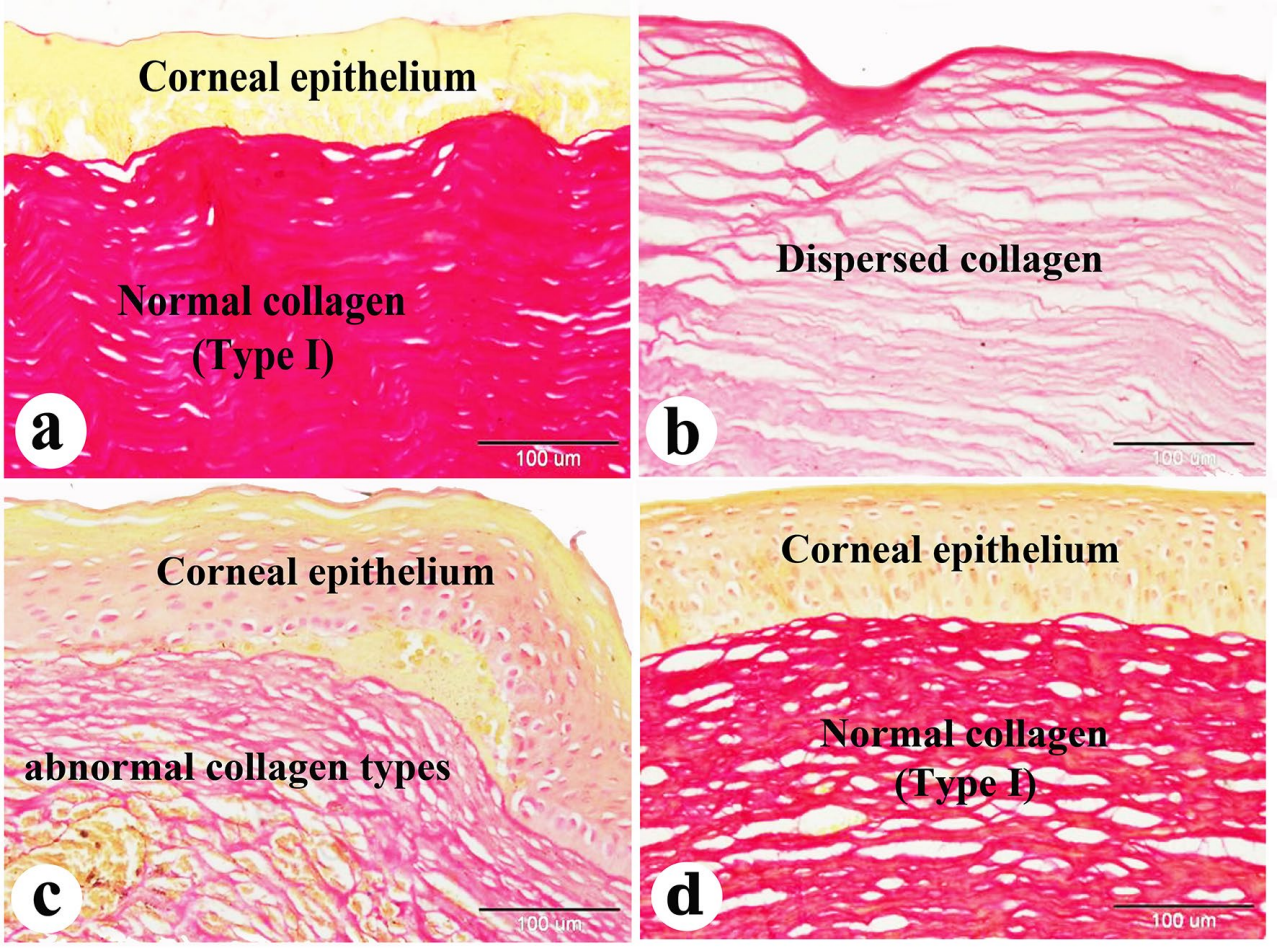
Photomicrograph of paraffin sections in donkey cornea. (**a**) The negative control group shows the normal histological structure of the cornea; Corneal epithelium and normal normal collagen type I. (**b**) Day 0 induced corneal ulcer group showing dispersed collagen type I. (**c**) The positive control group showing regenerated corneal epithelium and abnormal collagen types. (**d**) The PRF gel panel shows regenerated corneal epithelium and normal normal type I collagen. Note the new normal mature collagen stained red with the Picro-Sirius red staining technique. Original enlargement; ×200, scale bar = 100 m, Picro Sirius red spot technique.

**Figure 11:**
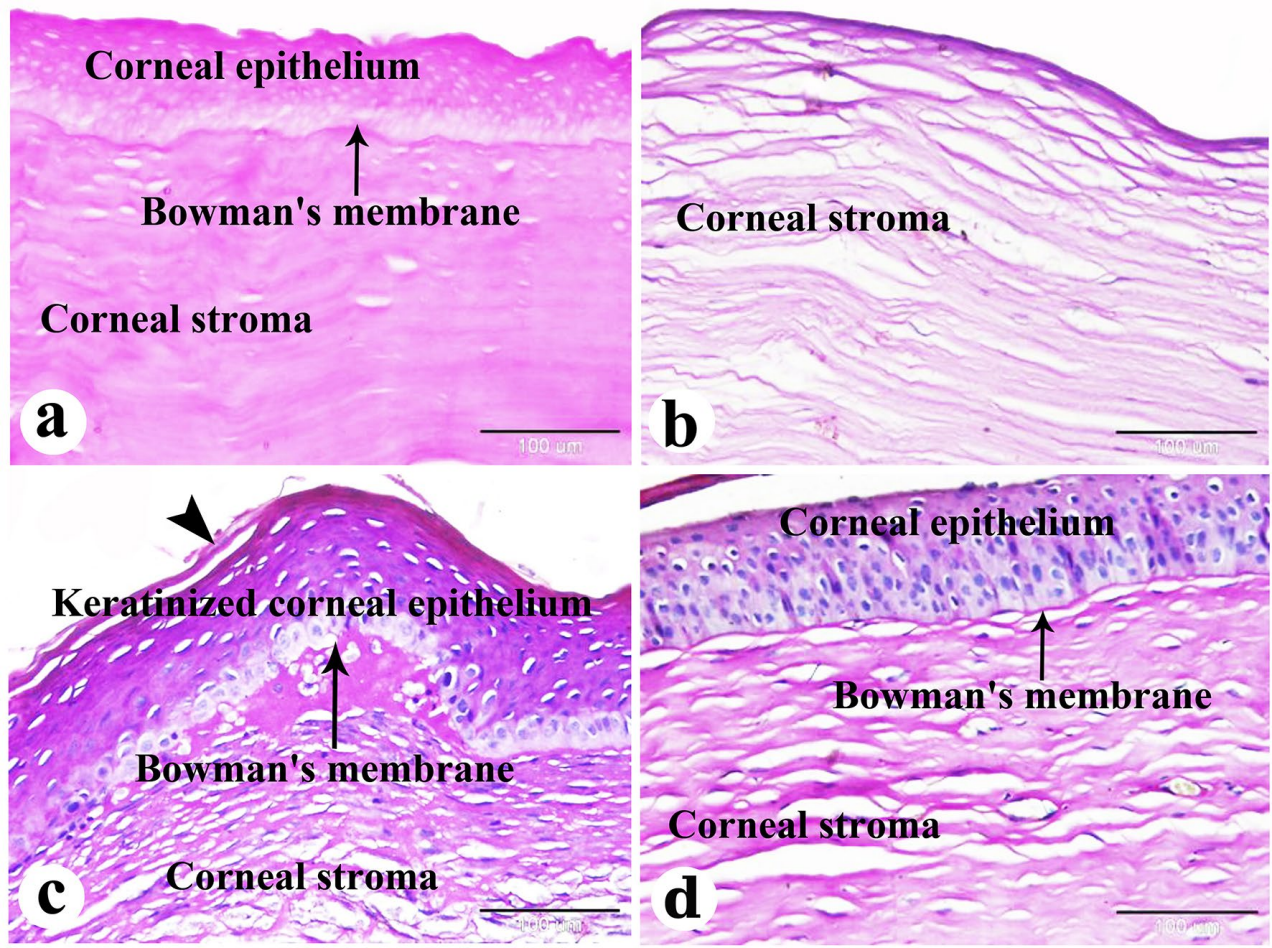
Photomicrograph of paraffin sections in donkey cornea. (**a**) The negative control group shows the normal histological structure of the cornea; Corneal epithelium, clear PAS-positive Bowman's membrane and PAS-positive collagen bundles of corneal stroma. (**b**) 0-day induced corneal ulcer group showing distributed faint PAS-positive collagen bundles of corneal stroma. (**c**) The positive control group, showing regenerated keratinized corneal epithelium, poorly clear and disrupted PAS-positive Bowman's membrane, and PAS-positive collagen bundles of the corneal stroma. Note the keratin layer (arrowhead) of the regenerated corneal epithelium. (**d**) The PRF gel panel showing regenerated corneal epithelium, clear PAS-positive Bowman's membrane, and PAS-positive collagen bundles of the corneal stroma. Original enlargement; ×200, scale bar = 100 m, PAS staining technique.

The negative control group exhibited a normal histological structure of the cornea, including the corneal epithelium, Bowman's membrane, corneal stroma, Descemet's membrane, and corneal endothelium (Figs. 6a, 7a, 8a, 9a, 10a and 11a).

On day 0, microscopic examination revealed epithelial loss and subepithelial stromal reaction characterized by edema and disorganized collagen fibers (Figs. 6b, 7b, 8b, 9b, 10b and 11b). In the positive control group, histopathological examination after 35 days showed re-epithelialization, stromal inflammation, vascularization, and thickening (Figs. 6c, 7c, 8c).

In contrast, the A-PRF gel group displayed re-epithelialization, stromal inflammation with normal collagen bundles, and regular keratocyte activation and proliferation (Figs. 6d, 7d, 8d). The present study demonstrated the activation and proliferation of keratocytes in the PRF gel group compared to the control group (Fig. 8a–d).

With Crossmons trichrome staining demonstrated irregular collagen bundles in the day 0-induced corneal ulcer group, dense irregular bundles in the positive control group, and dense, almost regular bundles in the PRF gel group (Fig. 9a–d). Picro Sirius red staining revealed dispersed type I collagen in the day 0-induced corneal ulcer group, abnormal collagen types in the positive control group and normal type I collagen in the PRF gel group (Fig. 10a–d).

PAS staining showed faint collagen bundles in the corneal stroma of the day 0-induced corneal ulcer group, regenerated keratinized epithelium and disrupted Bowman's membrane in the positive control group, and regenerated non-keratinized epithelium and intact Bowman's membrane in the PRF gel group (Fig. 11a–d). Overall, the histopathological changes and healing scores indicated improved healing in the PRF gel group compared to the control groups. (Table 3 provides a summary of these findings).

**Table 3:** Histopathological changes and healing score of corneal ulcers in donkeys:

Histopathological changes and repair events	Control	PRF gel
Edema	+++	+
Stromal disorganization	+++	+
Leucocytic infiltration	+++	+
Vascular congestion	+++	+
Pigmentary keratitis	+	+
Epithelial and stromal thickening	+++	+
Neovascularization	+++	+
Regular mature collagen replacement	+	++
Irregular fibrous tissue replacement	++	+
Fibroblasts activity	+ + +	+
Keratocytes activity	+	++
Epithelial regeneration	+++	++

## Discussion

Various degrees and intensities of keratitis were observed in both groups. The ulcer surface area, as measured by fluorescein staining, decreased significantly over time in all groups. Specifically, the A-PRF gel group showed a significant reduction in ulcer area. Histopathological examination revealed re-epithelialization of the corneal defect and a slightly decreased subepithelial stromal inflammatory response in the treated group. Additionally, there was evidence of keratocyte activation and proliferation in the A-PRF gel group compared to the control group.

To the author's knowledge, this study is the first to investigate the effects of A-PRF gel on corneal ulcer healing in horses. Previous studies have utilized PRF in the treatment of corneal ulcers in horses^32,43^, dogs^44–46^ and humans^47–50^. PRF can be used as a clot, membrane, or plug, either directly or after compression. The supernatant can also be used in drop^32^ or injectable (I-PRF) form^51^. In this study, A-PRF was used, which was a slower and more sustained release of growth factors as compared to standard PRF^52^. The fibrin network of A-PRF protects the growth factors from degradation, resulting in a higher and prolonged release of growth factors over 10 days^21,53^.

All donkeys in the study tolerated the experiment well, with only mild signs of stress observed four days after inducing corneal ulcers. Previous studies have also reported donkeys tolerating similar eye experiments without major complications^54^. Throughout the study period, there were no significant changes in the clinical symptoms of the donkeys, consistent with previous research ^47,54,55^.

However, ophthalmic signs of inflammation were observed in all donkeys after inducing corneal ulcers, particularly after A-PRF treatment. These signs were attributed to the irritating effect of sodium hydroxide used to induce the ulcers. Corneal alkaline burns are known to trigger a strong inflammatory response characterized by cellular infiltration and the production of enzymes and cytokines^56,57^. Similar signs of inflammation have been observed in other studies involving donkeys^58^ and rabbits^25,55^ in donkeys after chemically inducing corneal ulcers. Ocular symptoms were also observed during PRF treatment, as PRF can cause an inflammatory response^59^. It is worth noting that the administration of epidermal growth factor (EGF) in horses with corneal ulcers has been associated with adverse effects such as corneal edema, vascularization, melanosis, and scarring^43^. Similarly, rabbits exhibited a severe inflammatory reaction after PRF membrane application as a conjunctival transplant, although the intensity of the reaction decreased over time^47^. Prolonged episodes of corneal epithelialization have been suggested to increase collagenase production in the corneal stroma, potentially leading to corneal perforation^60^.

Towards the end of the study, the donkeys treated with A-PRF gel exhibited milder ocular symptoms compared to the control group. This can be attributed to the significant re-epithelialization of the corneal defect and the prolonged release of growth factors from the gel. The conjunctivitis observed in the A-PRF gel group may be due to the temporary tarsorrhaphy used to keep the gel in place. The study showed a significant decrease in the surface area of ​​the induced corneal ulcer over time in all animals, which is consistent with previous research in donkeys^58^. The inflammatory response following alkali combustion was quantified by area measurements of re-epithelialization and neovascularization^61^. Corneal wound healing is a complex process involving various factors, including proteinases, growth factors, cytokines, and lacrimal glands^62–64^ While PRF has been used to treat corneal ulcers and has shown potential benefits, other studies have reported adverse effects on ulcer healing^43,47,48^. In this study, A-PRF gel was directly applied to the ocular surface, which has been found to prevent dehydration^65^. Dehydration and shrinkage of the gel can affect its structural integrity, reduce growth factor content, and decrease leukocyte viability ^66^.

After applying Leukocyte Platelet Rich Fibrin (L-PRF), the corneal ulcer was completely sealed by the seventh day, with a whitish spot remaining until the 30th day. Although it weakened significantly by the 40th day, it was still present^44^. It is known that reducing the size of epithelial defects can lower the risk of infection and alleviate pain and discomfort43. Burling et al. (2000) found that re-epithelialization of corneal defects in horses started rapidly and progressed steadily within the first 5 to 7 days after surgery. However, the healing rate slowed down after this initial period^43^.

In the present study, all donkeys in both the control group and the A-PRF gel group showed no uptake of fluorescein dye at the end of the study, indicating successful re-epithelialization. Fluorescein dye is commonly used to diagnose corneal ulcers^67^, and treatment is continued until no fluorescein uptake is observed^68^. Although simple and indolent ulcers can be stained by fluorescein, in this study, staining was observed beneath the periphery of the corneal epithelium, suggesting detachment from the underlying stroma^12^. This resulted in larger surface values compared to concurrent values without staining.

To address corneal injuries, topical treatment with blood derivatives is utilized to compensate for the lack of natural angiogenesis in this avascular tissue. These blood-derived preparations contain growth factors, cytokines, and other signaling molecules that play a crucial role in cell turnover during corneal wound healing^69^. They can also suppress inflammation and possess antimicrobial properties^70^. The effectiveness of PRF in tissue regeneration is attributed to its key components, including fibrin as a supportive matrix, platelets rich in growth factors, and various types of leukocytes and stem cells that contribute to antibacterial, neovascularization, and regenerative properties^71,72^. The PRF membrane additionally provides mechanical support as a scaffold for cell proliferation, differentiation, and migration, which are essential for ocular surface regeneration^51^. Burling et al. (2000) studied the positive effects of growth factors like EGF and PDGF on the proliferation of epithelial cells and keratocytes^43^. Shen et al. (2011) have reported that the concentration and frequency of blood derivative administration have been found to influence the wound healing process. While the use of PRF gel is currently limited, it has shown promising results in the complete clinical healing of grade II corneal ulcers within 7 days^71^. However, incomplete healing was observed within 10 days for grade III ulcers, although a whitish patch remained^44^. In cases of severe ulcerative keratitis, PRF can be used as an alternative to corneal transplantation to preserve a significant portion of the cornea^44^.

In the present study, complications such as pigmentation and anterior synechia were observed in the PRF gel group, which is consistent with findings from other human studies^47,48^. However, the use of homologous L-PRF membrane proved effective in sealing severe corneal ulcers, including grade III ulcers with or without perforations and staphyloma^44^. Alkaline-induced corneal lesions often result in deep ulceration and perforation, leading to the formation of anterior synechiae and iris prolapse^72^. Dogs with corneal ulcers have also exhibited various clinical signs such as pain, inadequate tear film, corneal ulceration, edema, and significant neovascularization^56^. Alkali injuries are considered one of the most severe eye injuries, causing permanent visual impairment in one or both eyes. These injuries induce oxidative stress and inflammation in polymorphonuclear leukocytes^73^. The extent of inflammation following alkali burns can be measured by assessing the area of re-epithelialization and neovascularization^61^. Histopathological analysis revealed corneal epithelial regeneration in all donkeys in both the control and PRF gel groups. However, the control group exhibited signs of inflammation, including subepithelial coagulation, edema, leukocyte infiltration, vascular congestion, and neovascularization in the corneal stroma. In contrast, the PRF gel group only showed edema and neovascularization in the stroma. The PRF gel group also displayed numerous keratocytes between the stromal cells, resembling normal corneal histology.

PRF is known to have immune functions, including leukocyte chemotaxis and the release of cytokines such as IL-1, IL-4, IL-6, and TNF-a. It also contains anti-inflammatory cytokines like IL-4^74^. Microscopic examination revealed minimal inflammatory features in the gel-treated group compared to the control group, which is consistent with previous studies^72,75^. The presence of leukocytes and cytokines in the fibrin network may play a significant role in regulating inflammation and infection at the healing site^72,76^.

In this study, the use of PRF gel led to the proliferation of corneal epithelium and keratocytes, but some side effects were observed. Another study by Burling et al. (2000) found that the use of EGF in different doses resulted in faster healing of corneal ulcers, but also caused side effects such as corneal edema, vascularization, melanosis, and scarring^43^. The A-PRF gel promotes cellular proliferation, differentiation, and migration, providing temporary mechanical support to the growing cells and releasing growth factors slowly ^47,77^.

The corneal regeneration rate was higher in the group treated with A-PRF gel compared to the control group, likely due to the higher concentration of growth factors present in the gel ^47,76^. The results of this study showed that the stromal regeneration in the A-PRF gel group resembled that of normal corneal tissue, while the control group showed thickening of the corneal stroma. Another study by Can et al. (2016) using a PRF membrane for descemetocele treatment in a human patient also resulted in the synthesis of newly formed corneal tissue similar in thickness to the surrounding healthy cornea^47^. By Crossmons trichrome staining, the PRF gel group exhibited dense and regular collagen bundles in the stroma, whereas the control group had irregular and scattered collagen bundles. When stained with Picro Sirius red stain in the present study, the corneas in the A-PRF gel group showed mature type I collagen bundles, similar to normal corneal histology, while the control group exhibited abnormal collagen formation^47^. PAS staining revealed clear and intact Bowman's membrane and collagen bundles in the PRF gel group, resembling normal corneal histology. However, the control group showed disrupted Bowman's membrane. The control group also demonstrated keratinization in the regenerated corneal epithelium, which was not observed in the PRF-treated groups. Another study reported normal epithelial regeneration with a well-organized, non-keratinized epithelium, which was not observed in the PRF-treated groups^78^.

**Figure 12:**
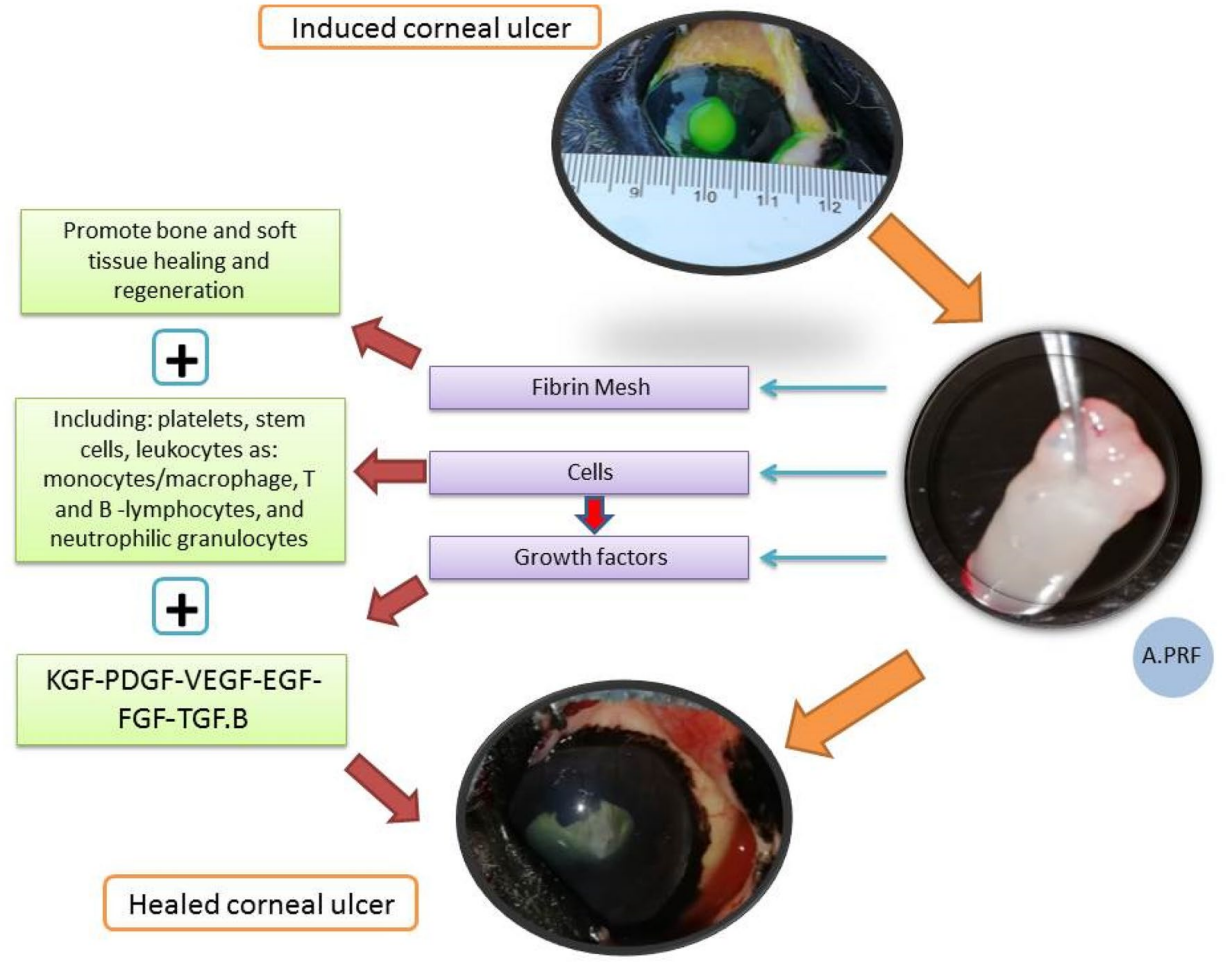
Diagram showing the overall mechanism of the A.PRF and the role of each components of it: KGF (keratinocyte growth factor): growth and new generation of keratinocytes. PDGF (platelet derived growth factor): cell growth, new generation and repair of blood vessels, collagen production. VEGF (vascular endothelial growth factor): growth and new generation of vascular endothelial cells. EGF (epidermal growth factor): promotion of epithelial cell growth, angiogenesis, and promotion of wound healing. FGF (fibroblast growth factor): tissue repair, cell growth, collagen production. TGF.B (transforming growth factor beta.1): growth of epithelial cells, endothelial cells, promotion of wound healing.

The overall mechanisms of the A.PRF illustrated in the following diagram (Fig. 12):

## Conclusion

Our findings indicate that advanced platelet-rich fibrin (A-PRF) is beneficial for the healing of corneal ulcers. The histological analysis showed that the PRF gel group had superior outcomes compared to the control group in terms of inflammation, re-epithelialization, absence of epithelial keratinization, regularity of collagen bundles, collagen type, and maturity. Additionally, the A-PRF gel group experienced fewer corneal complications during the healing process compared to the control group. These complications may be attributed to the repeated suturing of the eyelids and the irritation caused by directly applying the unfixed A-PRF gel to the cornea. Based on these results, we recommend using A-PRF gel as an alternative approach, avoiding eyelid suturing, and minimizing corneal irritation.

## Data Availability

All data generated or analyzed during this study are included in this published article and its additional information files.

## Abbreviations

PRF: Platelet rich fibrin
A-PRF: Advanced platelet rich fibrin
I-PRF: Injectable platelet rich fibrin
L-PRF: Leukocyte platelet rich fibrin
PRP: Platelet rich plasma
NaOH: Sodium hydroxide
EGF: Epidermal growth factor
PDGF: Platelet derived growth factor
KGF: Keratinocyte growth factor
VEGF: Vascular endothelial growth factor
FGF: Fibroblast growth factor
TGF.B: Transforming growth factor beta.1
BT: Body temperature
HR: Heart rate
RR: Respiratory rate
H&E: Haematoxylin and eosin
PAS: Periodic acid Schiff
